# Activity of alkanmonooxygenase alkB gene in strains of hydrocarbon-oxidizing bacteria isolated from petroleum products

**DOI:** 10.18699/VJGB-22-70

**Published:** 2022-10

**Authors:** T.N. Shapiro, N.A. Manucharova, E.S. Lobakova

**Affiliations:** Lomonosov Moscow State University, Faculty of Biology, Moscow, Russia; Lomonosov Moscow State University, Faculty of Soil Science, Moscow, Russia; Lomonosov Moscow State University, Faculty of Biology, Moscow, Russia

**Keywords:** biodamage, petroleum products, hydrocarbon-oxidizing bacteria, biodegradation, alkanmonooxygenase, alkB gene, real-time PC, биоповреждение, нефтепродукты, углеводородокисляющие бактерии, биодеградация, алканмонооксигеназы, ген alkB, ПЦР в реальном времени

## Abstract

Alkanmonooxygenase enzymes AlkB and Cyp153 are responsible for the aerobic degradation of n-alkanes of petroleum and petroleum products. To prove the usage of n-alkanes from oil and petroleum products by hydrocarbon-oxidizing bacteria isolated from aviation kerosene TS-1 and automobile gasoline AI-95, the detection of the key genes alkB, Alk1, Alk2, Alk3 and Cyp153 encoding alkanmonooxygenases AlkB and Cyp153 (responsible for the oxidation of hydrocarbons with a certain chain length) was carried out. It was found that bacterial strains isolated from TS-1 jet fuel, except Deinococcus sp. Bi7, had at least one of the studied n-alkane degradation genes. The strains Sphingobacterium multivorum Bi2; Alcaligenes faecalis Bi3; Rhodococcus sp. Bi4; Sphingobacterium sp. Bi5; Rhodococcus erythropolis Bi6 contained the alkB gene. In the strains of hydrocarbon-oxidizing bacteria isolated from gasoline AI- 95, this alkanmonooxygenase gene was not detected. Using the real-time PCR method, the activity of the alkB gene in all bacterial strains isolated from petroleum products was analyzed and the number of its copies was determined. By real-time PCR using a primer with a different sequence of nucleotides to detect the alkB gene, its activity was established in all bacterial strains isolated from gasoline AI-95; besides, the strain Paenibacillus agaridevorans Bi11 was assigned to the group with a high level of its activity (1290 copies/ml). According to the assessment of the growth of isolated hydrocarbon-oxidizing bacteria on a solid Evans mineral medium with the addition of the model mixture of hydrocarbons, the strains were divided into three groups. The distributions of strains of hydrocarbon-oxidizing bacteria in the groups based on the activity of the alkB gene and groups formed based on the growth ability and use of the model mixture of hydrocarbons and petroleum products were found to be consistent. The results obtained indicate that we need to use a complex of molecular and physiological methods for a comprehensive analysis of the distribution of the studied genes in bacteria and to assess their activity in the strains of hydrocarbon-oxidizing bacteria capable of biodegradation of petroleum hydrocarbons.

## Abstract (ru)

Ферменты алканмонооксигеназы AlkB и Cyp153 ответственны за аэробную деградацию н-алканов нефти и нефтепродуктов. Для доказательства использования штаммами углеводородокисляющих бактерий, выделенных из авиационного керосина ТС-1 и автомобильного бензина АИ-95, н-алканов нефти и нефтепродуктов, проведена детекция ключевых генов alkB, Alk1, Alk2, Alk3 и Cyp153, кодирующих алканмонооксигеназы AlkB и Cyp153, ответственных за окисление углеводородов с определенной длиной цепи. Установлено, что штаммы бактерий, изолированные из реактивного топлива ТС-1, за исключением Deinococcus sp. Bi7, имели как минимум один из исследованных генов деградации н-алканов. Штаммы Sphingobacterium multivorum Bi2, Alcaligenes faecalis Bi3, Rhodococcus sp. Bi4, Sphingobacterium sp. Bi5, Rhodococcus erythropolis Bi6 содержали ген alkB. У штаммов углеводородокисляющих бактерий, выделенных из бензина АИ-95, этот ген алканмонооксигеназы не был детектирован. С помощью метода ПЦР в реальном времени проанализирована активность гена alkB у всех полученных из нефтепродуктов штаммов бактерий и определено число его копий. Методом ПЦР в реальном времени с использованием праймера с другой последовательностью нуклеотидов для детек- ции гена alkB установлена его активность у всех штаммов бактерий, выделенных из бензина АИ-95, причем штамм Paenibacillus agaridevorans Bi11 отнесен к группе с высоким уровнем его активности (1290 копий/мл). По оценке роста исследованных углеводородокисляющих бактерий на плотной минеральной среде Эванса с мо- дельной смесью углеводородов штаммы были разделены на три группы. Отмечены совпадения результатов по распределению штаммов углеводородокисляющих бактерий в группах по активности гена alkB и группах, сформированных на основе способности роста и использования модельной смеси углеводородов и нефте- продуктов. Полученные результаты свидетельствуют о необходимости применения комплекса молекулярно- генетических и физиологических методов для всестороннего анализа распространения исследуемых генов у бактерий и оценки их активности в штаммах углеводородокисляющих бактерий, способных к биодеградации углеводородов нефтепродуктов.

## Introduction

Petroleum products are the main source of energy from the economical point of view and in human life. Data about biological contamination of petroleum products and, first of all, various types of fuels, especially aviation kerosene, has recently increased significantly in the open press (Martin- Sanchez et al., 2018). Direct and indirect losses from microbiological corrosion of petroleum products in industrialized countries range from 2 to 5 % of the annual gross domestic product (Karimova, 2007). The study of the ability of strains of hydrocarbon-oxidizing bacteria isolated from petroleum products to use n-alkanes plays an important role both for protecting petroleum products from bio-damage and in the application of these strains for the disposal of emergency oil spills in water areas and on land (Dedov et al., 2017). In addition, the ability of bacteria to assimilate petroleum hydrocarbons can be the reason for the loss of their quality during transportation, storage and usage of equipment (Martin- Sanchez et al., 2018).

As a rule, microorganisms are capable of selective assimilation of certain types of hydrocarbons, which is determined by the number of carbon atoms and the peculiarity of the structure of the hydrocarbon. In natural conditions, microorganisms form communities in which a single chain of oxidation of hydrocarbons of oil and petroleum products is formed by the type of metabiosis. Each microorganism of the community, having specific enzyme systems aimed at using a certain type of hydrocarbons, uses this substrate in its metabolism. Therefore, with the joint action of microorganisms of the community, not only a larger amount, but also a wider range of hydrocarbons of oil and petroleum products is used (Timergazina, Perekhodova, 2012).

It is known that the vast majority of bacterial transformations of hydrocarbons are oxidative reactions that occur most actively in aerobic conditions. There are data on the molecular mechanisms and ways of aerobic biodegradation of hydrocarbons, which are as follows: 1) many multi-purpose oxygenase systems forming active complexes with hydrocarbon substrates and molecular oxygen have been discovered; 2) several enzymes involved in the initial stage of aerobic biodegradation of alkanes have been characterized (Coon, 2005; Funhoff et al., 2006; Van Beilen, Funhoff, 2007); 3) the metagenomic approach has made it possible to describe new metabolic pathways of hydrocarbon degradation, different from those previously characterized in cultured pure bacterial strains (Sierra-García et al., 2014) and 4) new phylotypes of alkanmonooxygenase (alkB) genes encoding alkanmonooxygenases have been found in marine ecosystems (Wasmund et al., 2009; Smith et al., 2013).

Aerobic degradation of alkanes can be carried out by two main types of enzymes: alkanmonooxygenase AlkB (also known as alkanhydroxylase) and some cytochrome P450 systems (Van Beilen et al., 2006) found in bacteria of the genera Pseudomonas (Johnson, Hyman, 2006), Rhodococcus (Sameshima et al., 2008), Acinetobacter (Throne-Holst et al., 2007), Alcanivorax (Liu, Shao, 2005), Burkholderia (Mohanty, Mukherji, 2008), Geobacillus (Vomberg, Klinner, 2000) and Gordonia (Kato et al., 2009). Genes encoding the protein complex of alkanmonooxygenase CYP 153 P450 have been studied by several authors (Whyte et al., 1998; Smits et al., 1999; Kloos et al., 2006; Powell et al., 2006), molecular methods for their identification have been proposed not only in pure cultures, but also at the level of the microbial community (Wang et al., 2010).

However, the regulation of the expression of genes encoding the degradation pathways of alkanes still has many unresolved issues, due to the fact that in many cases genes of central metabolism also participate in these processes (Paisse et al., 2011). In addition, since these genes and their products are adaptive, many of them are often located in plasmids, which can contribute to their variability and horizontal transfer (Korshunova et al., 2011).

The cytochrome P450 Cyp153 family is a type of alkanmonooxygenases used for the degradation of short-chain and medium-chain n-alkanes and are commonly found in hydrocarbon-oxidizing bacteria lacking AlkB monooxygenases (Van Beilen, Funhoff, 2007). Oxygen-activated systems lacking this cytochrome are characteristic of prokaryotes and are formed by another integral membrane-bound monooxygenase encoded in most bacteria by the alkB gene, and electron transport proteins such as rubredoxin and NADH-dependent reductase encoded by the alkG and alkT genes, respectively (Van Beilen et al., 2006; Cappelletti et al., 2011). AlkB monooxygenase has been detected in bacteria of various systematic groups and is used by them for oxidation of n-alkanes with a chain length up to C16 (Wasmund et al., 2009). Thus, Alklike genes have been studied in Gram-positive bacteria such as Rhodococcus, Mycobacterium, Nocardia and Praserella (Andreoni et al., 2000; Vomberg, Klinner, 2000; Van Beilen et al., 2002; Whyte et al., 2002).

To confirm the presence of a specific n-alkane oxidation system and the homology degree of its sequence with the previously studied sequences of the alkB gene, the method of amplification of fragments of the alkB gene using specific primers for this gene was mainly used. Studies on the genetic and structural organization of n-alkane oxidation systems, regulation of their genes and the spectrum of utilized substrates were carried out only for individual strains. It should be considered that each microorganism has a certain set of inducible oxygenase systems and the ability to degrade some hydrocarbons depends on the expression of the corresponding oxygenase (Redmond et al., 2010).

The detection and determination of the activity of key genes responsible for the oxidation of certain types of hydrocarbons in oil and petroleum products is a direct proof of the use of hydrocarbons by hydrocarbon-oxidizing bacteria, and can also serve as a measure of the assessment of the metabolic activity of a particular microorganism.

The aim of the work was to detect alkB, Alk1, Alk2, Alk3 and Cyp153 genes encoding AlkB and Cyp153 alkanmonooxygenases in strains of hydrocarbon-oxidizing bacteria isolated from samples of TS-1 jet fuel and AI-95 gasoline, and to study the activity of the alkB gene by real-time PCR.

## Materials and methods

Objects of research. In the current study, 13 strains of hydrocarbon- oxidizing bacteria isolated from TS-1 jet fuel and AI-95 gasoline (Shapiro et al., 2021) were used. The sequences of the fragment of the 16S rRNA gene of isolated strains of hydrocarbon-oxidizing bacteria are deposited in the Genbank international database (Table 1). Bacterial strains are stored in the collection of the Department of Bioengineering of the Faculty of Biology of Moscow State University. The cultures were maintained on a solid organic Rich medium containing peptone, yeast extract, casein hydrolysate, and glucose (Lysak et al., 2003), the growth of isolated strains in the presence of petroleum products was analyzed on an Evans mineral medium (Evans et al., 1970) with the addition of hydrocarbons as the only carbon source.

**Table 1.**
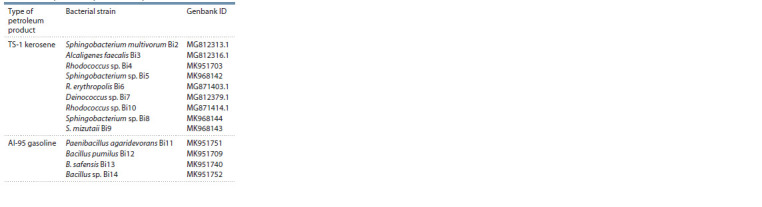
Strains of hydrocarbon-oxidizing bacteria isolated from petroleum products samples

Isolation of bacterial DNA. DNA isolation was carried out after 7 days of cultivation of hydrocarbon-oxidizing bacteria strains on the Rich medium. To isolate bacterial DNA, the Thermo Scientific™ MagJET™ Plant Genomic DNA Kit was used as described earlier (Shapiro et al., 2021).

Assessment of the growth of pure cultures of hydrocarbon- oxidizing bacteria on a medium with model hydrocarbons. The growth of isolated cultures of hydrocarbonoxidizing bacteria in the presence of hydrocarbons was compared using the M.V. Zhurina et al. (2008) method. 0.025 μl of culture suspension of a hydrocarbon-oxidizing bacteria strain with an optical density (OD) of 0.2 was added onto the solid EM medium containing 1.96 % by volume of a mixture of hydrocarbons No. 1 (С15Н32, С16Н34, С18Н38 and С9Н12-pseudocumol) and distributed over the surface of the Petri dish with a spatula. After 7 days, the grown colonies of microorganisms were washed off with a 1 % NaCl solution in two portions of 5 ml. In the combined sample, the optical density of the obtained cell suspension was measured using the spectrophotometer КFК-2-UHL 4.2 at λ = 540 nm and the thickness of the optical layer l = 10 mm.

Detection of alkanmonooxygenase genes alkB, Alk1, Alk2, Alk3 and Cyp153. To obtain the PCR products of genes encoding various alkanmonooxygenases (Kohno et al., 2002; Ivanova et al., 2014) (the sequences of the used primers are shown in Table 2), PCR was performed with the genomic DNA of the isolated strains using the following parameters: initiation – 94 °C × 3 min, subsequent 35 cycles – 94 ° C × 30 s, 55 °C (Cyp153) or 60 °C (alkB) × 40 C, 72 ° C – 1 min; final polymerization – 72 ° C × 7 min. (Ivanova et al., 2014). For genes Alk1-3 PCR was performed in the following mode: initial initiation – 94 °C × 3 min, subsequent 30 cycles – 94 °C × 60 s, 40 °C × 30 s, 72 °C – 30 s; final polymerization – 72 °C × 7 min (Kohno et al., 2002).

**Table 2.**
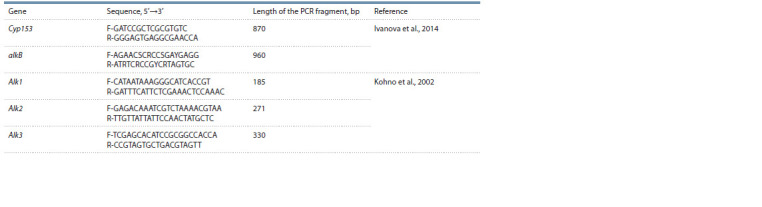
Sequences of primers used for the detection of alkB, Alk1, Alk2, Alk3 and Cyp153 genes encoding alkanmonooxygenases

PCR was performed on a Mastercycler Gradient DNA amplifier (Eppendorf, Germany). The volume of the amplification mixture was 50 μl and had the following composition: 10 ml of 1× Taq polymerase buffer (Evrogen, Russia), 1 ml of forward and reverse primers, 1 ml of DNA of the sample and 37 ml of water. The amplification results were recorded using electrophoresis. The PCR purification of the product was carried out using the Cleanup Standard kit (Eurogen).

Real-time PCR. The real-time PCR method was used to quantify the number of DNA copies containing the functional alkB gene responsible for the degradation of n-alkanes. The measurement was carried out on a DTLite4 (DNA Technology, Russia) amplifier after 7 days of cultivation of hydrocarbonoxidizing bacteria strains on Rich medium (Lysak et al., 2003), according to the method described in (Manucharova et al., 2021). Sequences of primers used to identify hydrocarbonoxidizing bacteria strains with the functional gene alkB were as follows: F(TGGCCGGCTACTCCGATGATCGGAATCT GG); R(CGCGTGGTGATCCGAGTGCCGCTGAAGGTG) (Whyte et al., 2002).

The amount of DNA under study was expressed in absolute or relative units. Quantitative determination of the DNA matrix was carried out in the presence of three standards and negative control (a sample without a DNA matrix).

## Results and discussion

Previously, strains of hydrocarbon-oxidizing bacteria were isolated from contaminated samples of petroleum products (TS-1 jet fuel and AI-95 gasoline), identified and characterized (Shapiro et al., 2021). 9 strains of hydrocarbon-oxidizing bacteria were isolated, described and identified from TS-1 fuel, and 4 strains were isolated from AI-95 gasoline.

All isolated strains of hydrocarbon-oxidizing bacteria were analyzed for the presence of genes encoding alkanmonooxygenases: alkB, Cyp153, Alk1, Alk2 and Alk3 (Table 3). The Alk1 gene encodes alkanmonooxygenase AlkB, which catalyzes the reactions of terminal oxidation of n-alkanes with a chain length of С6–С12 in representatives of the Pseudomonas genus. The Alk2 gene encodes alkanmonooxygenase AlkB in representatives of the Acinetobacter genus, which catalyzes the reactions of terminal oxidation of n-alkanes with a chain length > C12 using monooxygenases or dioxygenases. The Alk3 gene encodes alkanmonooxygenase AlkB, which has substrate specificity to n-alkanes and oxidase systems (Kohno et al., 2002).

**Table 3.**
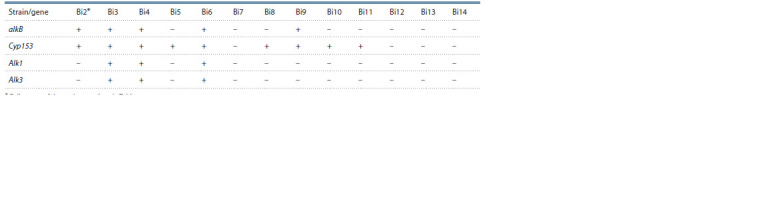
The presence of the studied alkanmonooxygenase genes for the oxidation of hydrocarbons alkB, Cyp153, Alk1, Alk2 and Alk3 in strains of hydrocarbon-oxidizing bacteria isolated from petroleum products * Full names of the strains are given in Table 1.

**Fig. 1.**
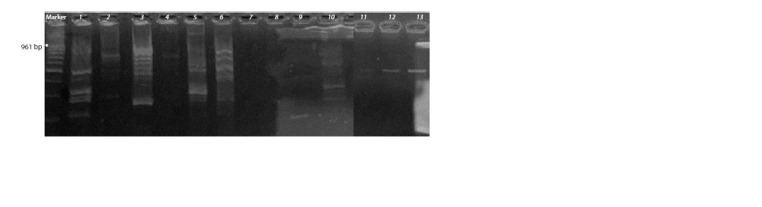
Electrophoresis in agarose gel of the PCR product of the alkB gene. 1 – Sphingobacterium multivorum Bi2; 2 – Alcaligenes faecalis Bi3; 3 – Rhodococcus sp. Bi4; 4 – Sphingobacterium sp. Bi5; 5 – Rhodococcus erythropolis Bi6; 6 – Deinococcus sp. Bi7; 7 – Sphingobacterium sp. Bi8; 8 – Sphingobacterium mizutaii; 9 – Rhodococcus sp. Bi10; 10 – Bacillus pumilus Bi12; 11 – Bacillus safensis Bi13; 12 – Bacillus sp. Bi14; 13 – Paenibacillus agaridevorans Bi11. DNA length marker (100 + bp DNA Ladder). * – is the expected length of the target PCR product.

It was found that the alkanmonooxygenase Alk2 gene, typical mainly for Acinetobacter bacteria (Kohno et al., 2002), is absent in all bacterial strains studied. Among the strains isolated from TS-1 jet fuel, the strain Deinococcus sp. Bi7 did not contain the studied alkanmonooxygenase genes. All the other strains isolated from TS-1 fuel had at least one of the studied n-alkane degradation genes. Five strains (Sphingobacterium multivorum Bi2, Alcaligenes faecalis Bi3, Rhodococcus sp. Bi4, Sphingobacterium sp. Bi5, Rhodococcus erythropolis Bi6) had the alkB gene. In the strains of hydrocarbon-oxidizing bacteria isolated from gasoline AI-95, this alkanmonooxygenase gene was not detected (Fig. 1, see Table 3)

All the studied alkanmonooxygenase genes – alkB, Cyp153, Alk1 and Alk3 were identified in the strains A. faecalis Bi3, Rhodococcus sp. Bi4 and R. erythropolis Bi6. It is interesting that different isoforms of the alkB gene and the Cyp153 gene were simultaneously present in these bacteria, and genes alkB, Cyp153 – in the strains of Sphingobacterium multivorum Bi2 and S. mizutaii Bi9. According to the resent data, the enzyme Cyp153 is a type of alkanmonooxygenase involved in the degradation of short-chain and medium-chain n-alkanes in hydrocarbon-oxidizing bacteria that do not have alkB alkanmonooxygenases (Van Beilen, Funhoff, 2007).

n-alkanes account for up to 88 % of the volume in natural oil and petroleum products and can serve as an energy source for microorganisms capable of decomposing them (Van Beilen et al., 2003; Dedov et al., 2017). The detection of alkanmonooxygenase group genes was previously carried out for bacterial communities isolated from petroleum products, and the activity of strains against the degradation of various hydrocarbons, including n-alkanes, was shown (Likhoshvay et al., 2014; Lomakina et al., 2014).

alkB family genes are usually present in the genomes of both gram-positive and gram-negative bacteria in several variants (Van Beilen et al., 2003). This is consistent with the data obtained by us on the presence of several alkB family genes in isolated strains of gram-negative bacteria of the Sphingobacterium genus and gram-positive bacteria of the Rhodococcus genus.

The ability to degrade n-alkanes in strains for which this has not been described in the literature before may be evidence of the gene localization in the plasmid and its horizontal transfer between community members, which was shown in the works of T.P. Turova et al. (2008), where bacteria of the Geobacillus genus could acquire alkB genes from bacteria of the Rhodococcus genus.

Among the strains of hydrocarbon-oxidizing bacteria isolated from AI-95 gasoline, only the Cyp153 gene was detected in P. agaridevorans Bi11.

**Fig. 2.**
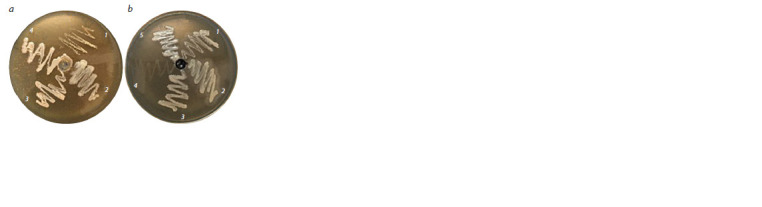
Growth of the several studied cultures on the Evans mineral medium in the presence of TS-1 jet fuel (a) and oil (b). (а) 1 – Sphingobacterium multivorum Bi2; 2 – Sphingobacterium sp. Bi5; 3 – Rhodococcus sp. Bi4; 4 – Alcaligenes faecalis Bi3; (b) 1 – Rhodococcus sp. Bi4; 2 – Sphingobacterium mizutaii Bi9; 3 – Sphingobacterium sp. Bi8; 4 – Deinococcus sp. Bi7; 5 – Rhodococcus erythropolis Bi6.

**Fig. 3.**
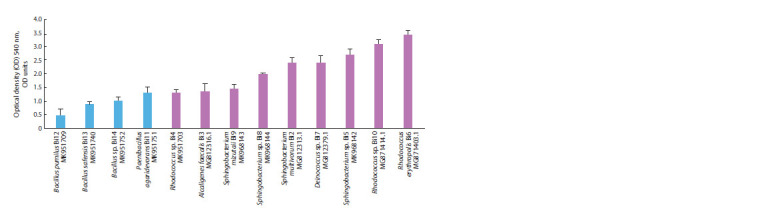
Growth of isolated bacterial strains from AI-95 gasoline (blue columns) and TS-1 jet fuel (pink columns) on a solid medium with a mixture of hydrocarbons No. 1 for seven days.

The data on the presence of alkB family genes in the studied bacterial strains only partially agreed with the data on their ability to grow on liquid and solid media in the presence of 1 % n-alkanes with different carbon chains length (Shapiro et al., 2021). Thus, strains Sphingobacterium mizutaii Bi9, Bacillus pumilus Bi12; Bacillus safensis Bi13; Bacillus sp. Bi14; Paenibacillus agaridevorans Bi11 grew on a model mixture of hydrocarbons containing alkanes with different chain lengths, TS-1 fuel and oil (Fig. 2 and 3). Also, in some cases, the ability to grow and the high activity of the isolated strains in degrading n-alkanes of the model hydrocarbon mixture in the absence of this gene were established (see Fig. 3).

Growth evaluation of hydrocarbon-oxidizing bacteria pure cultures on a solid EM medium with a model mixture of hydrocarbons (see Fig. 3) allowed us to divide the strains by growth rate into three groups. This division was proposed by us and is based on the following: group 1 (active cultures) – the value of the optical density of the cell suspension after cultivation for 7 days from 3 units and above; group 2 (medium activity) – from 2 to 3 units; group 3 (low activity) – the value of the optical density of the cell suspension less than 2 units.

It was found that the most active group of strains capable of using a model mixture of hydrocarbons included strains R. erythropolis Bi6, Rhodococcus sp. Bi10. The average growth rate is typical for strains Deinococcus sp. Bi7, Sphingobacterium sp. Bi5, S. multivorum Bi2 and Sphingobacterium sp. Bi8. At the same time, the tested alkanmonooxygenase genes were not detected in the strain Deinococcus sp. Bi7. The strains Rhodococcus sp. Bi4, S. mizutaii Bi9, Ochrobactrum sp. Bi1 and A. faecalis Bi3 and all strains isolated from gasoline had the slowest growth in the presence of a model mixture of hydrocarbons. At the same time, bacterial strains isolated from gasoline AI-95 – Bacillus safensis Bi13; Bacillus sp. Bi14, in which the alkB gene was not detected, used pentadecane, octadecane and hexadecane of a model mixture by more than 80 % (Shapiro et al., 2021). In this regard, a quantitative analysis of the number of DNA copies containing the functional alkanmonooxygenase gene in all isolated strains of hydrocarbon-oxidizing bacteria was carried out. Based on the results of real-time PCR, it was found that the alkB gene is present and active in all bacterial strains isolated from petroleum products.

According to the number of copies of the gene, all bacterial strains were divided into two groups: the first group with the highest activity of the alkB gene, for which the concentration values ranged from 1290 to 8060 DNA copies/ml, and the second group, where the concentration values were from 10.4 to 786 DNA copies/ml:

I group Alcaligenes faecalis Bi3 Sphingobacterium multivorum Bi2 Sphingobacterium mizutaii Bi9 Sphingobacterium sp. Bi5 Paenibacillus agaridevorans Bi11

II group Rhodococcus sp. Bi4 Rhodococcus erythropolis Bi6 Rhodococcus sp. Bi10 Sphingobacterium sp. Bi8 Deinococcus sp. Bi7 Bacillus pumilus Bi12 Bacillus safensis Bi13 Bacillus sp. Bi14

It was found that all strains of hydrocarbon-oxidizing bacteria isolated from gasoline AI-95 showed the activity of the alkB gene, and the strain Paenibacillus agaridevorans Bi11 was assigned to the first group of strains with a high level of its activity (1290 DNA copies/ml). The results obtained were consistent with the data on the ability of strains isolated from petroleum products to grow (see Fig. 3) and use hydrocarbons of a model mixture of hydrocarbons (Shapiro et al., 2021). There were also coincidences of the results on the distribution of strains of hydrocarbon-oxidizing bacteria in groups based on the activity of the alkB gene (see Table 2) and groups formed on the basis of their growth ability and the use of a model mixture of hydrocarbons and petroleum products (Shapiro et al., 2021).

In bacteria growing on petroleum products, including both short-chain and long-chain n-alkanes, their oxidation system includes several isoenzymes of the key protein alkanmonooxygenase. The strains of bacteria isolated from TS-1 jet fuel and AI-95 gasoline are capable of using a wide range of substrates, which suggests that they have a complex alkanmonooxygenase system. It has been established that representatives of different groups of hydrocarbon destructor microorganisms may have several evolutionary variants of alkanmonooxygenase enzymes, which requires the selection of primer sets for different hydrocarbon-oxidizing bacteria that allow the identification of all variants of hydrocarbon oxygenase genes. In such cases, it is proposed to apply several variants of primers to different groups of isoenzymes (Kohno et al., 2002; Heiss-Blanquet et al., 2005). In our work, two types of primers were used to detect the presence and activity of the alkB gene. The detection of the alkB gene with primers proposed in the article by A.E. Ivanova and co-authors (2014) showed the presence of this gene in five bacterial strains, and with primers by L.G. Whyte and co-authors (2002) – in all studied strains of petroleum products destructors. This may indicate the greater versatility of the primers proposed by L.G. Whyte and co-author (2002), on the one hand, or the presence of a specific isoform of the enzyme, on the other.

## Conclusion

Thus, real-time PCR revealed the activity of the alkB gene in all strains of hydrocarbon-oxidizing bacteria isolated from TS-1 jet fuel and AI-95 gasoline. A significant quantitative difference in the activity of this gene in the isolated strains was shown. For strains isolated from gasoline, the activity data correspond to physiological and biochemical data on bacterial growth in the presence of a model mixture of hydrocarbons and the efficiency of their degradation (Shapiro et al., 2021). The results obtained indicate the need to use a set of methods (a polyphase approach) for a comprehensive assessment of the ability of hydrocarbon-oxidizing bacteria strains to degrade petroleum hydrocarbons, including the usage of molecular (in particular, PCR) and physiological methods to analyze the distribution and homology of the specific studied gene in bacteria.

## Conflict of interest

The authors declare no conflict of interest.
